# Supplementary material for: Exploring physics of ferroelectric domain walls via Bayesian analysis of atomically resolved STEM data
Source: Nat Commun. 2020 Dec 11;11:6361. doi: 10.1038/s41467-020-19907-2 (PMC7733522; doi:10.1038/s41467-020-19907-2)
Supplement: Supplementary file 1 — Supplementary Information [file 41467_2020_19907_MOESM1_ESM.pdf]

Supplementary Information:

Exploring physics of ferroelectric domain walls via Bayesian analysis of atomically resolved STEM data

Christopher T. Nelson,[1] Rama K. Vasudevan,[1] Xiaohang Zhang,[2] Maxim Ziatdinov,[1] Eugene A. Eliseev,[3] Ichiro Takeuchi,[2] Anna N. Morozovska,[4] and Sergei V. Kalinin[1,*]

[1] The Center for Nanophase Materials Sciences, Oak Ridge National Laboratory, Oak Ridge, TN 37831

[2] Department of Materials Science and Engineering, University of Maryland, College Park, MD 20742

[3] Institute for Problems of Materials Science, National Academy of Sciences of Ukraine, Krjijanovskogo 3, 03142 Kyiv, Ukraine

[4] Institute of Physics, National Academy of Sciences of Ukraine, 46, pr. Nauky, 03028 Kyiv, Ukraine

*email: sergei2@ornl.gov

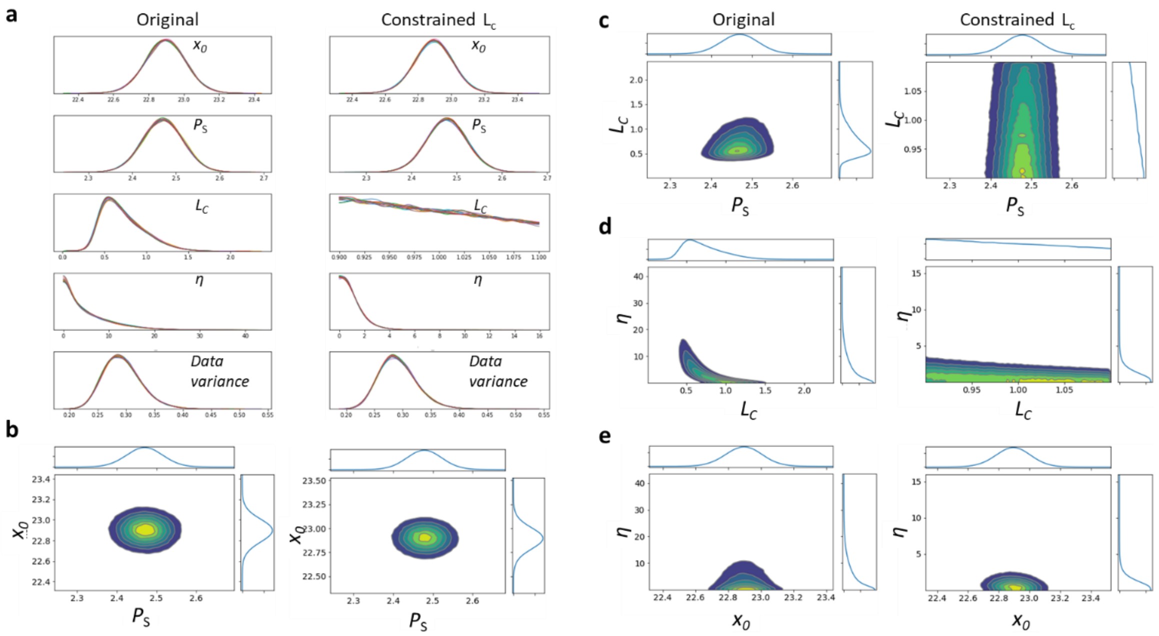

Supplementary Figure 1. **Effect of additional L_C constraint for 109° domain wall model II.** (a) Posterior probability densities for the model parameters: wall position (x_0), saturation polarization (P_S), correlation length (L_C), η, and the data variance for the original bounds (left) and with $0.9 < L_C < 1.1$ (right). (b-e) are selected 2D joint probability densities for parameter combinations (b) x_0-P_S, (c) L_C-P_S, (d) η- L_C, and (e) η- L_c, shown as a pair with the original bounds at left, and constrained L_C at right.

Supplementary Methods: Fitting functions for ferroelectric domain wall profiles, Ginzburg-Landau-Devonshire (LGD) approach

In the classical Ginzburg-Landau-Devonshire (LGD) approach of multiferroic materials, the relationship between some of these variables can be found via the minimization of the free energy functional. In the most general form, two vectorial long-range order parameters, polarization components P_i and oxygen octahedral tilts Φ_i , will be used for the description of the antiferrodistortive (AFD), ferroelectric (FE), and antiferroelectric (AFE) long-range orders in the rare-earth (RE = Sm, La, Pr, Eu, etc. [1, 2, 3, 4]) doped $BiFeO_3$. The bulk part of LGD thermodynamic potential consists of the following contributions:

$$G = \int d^3x\left(\Delta G_{AFD} + \Delta G_{FE} + \Delta G_{BQC} + \Delta G_{ST} + \Delta G_{EL}\right). \quad \text{(SI.1)}$$

The compact form of the AFD contribution is [5]:

$$\Delta G_{AFD} = b_i\Phi_i^2 + b_{ij}\Phi_i^2\Phi_j^2 + b_{ijk}\Phi_i^2\Phi_j^2\Phi_k^2 + v_{ijkl}\frac{\partial\Phi_i}{\partial x_k}\frac{\partial\Phi_j}{\partial x_l}. \quad \text{(SI.2a)}$$

We assume that the coefficients b_i are temperature dependent in accordance with a Barrett law [6], $b_i(T) = b_T T_{q\Phi}\left[\coth\left(T_{q\Phi}/T\right) - \coth\left(T_{q\Phi}/T_{\Phi}\right)\right]$, where T_{Φ} is the AFD transition temperature and $T_{q\Phi}$ is a characteristic temperature. Numerical values of the phenomenological coefficients b_i, b_{ij}, b_{ijk} and gradient coefficients v_{ij} included in Eq.(SI.2a) are found in Supplementary Table 1.

The compact form of the FE and AFE contributions are:

$$\Delta G_{FE} = a_i(P_i^2 + A_i^2) + a_{ij}\left(P_i^2P_j^2 + A_i^2A_j^2\right) + a_{ijk}\left(P_i^2P_j^2P_k^2 + A_i^2A_j^2A_k^2\right) + \gamma_{ij}^{ab}\left(P_iP_j - A_iA_j\right) + g_{ijkl}^{aa}\left(\frac{\partial P_i}{\partial x_k}\frac{\partial P_j}{\partial x_l} + \frac{\partial A_i}{\partial x_k}\frac{\partial A_j}{\partial x_l}\right) + g_{ijkl}^{ab}\left(\frac{\partial P_i}{\partial x_k}\frac{\partial P_j}{\partial x_l} - \frac{\partial A_i}{\partial x_k}\frac{\partial A_j}{\partial x_l}\right), \quad \text{(SI.2b)}$$

where the FE and AFE order parameters, $P_i = \frac{1}{2}\left(P_i^a + P_i^b\right)$ and $A_i = \frac{1}{2}\left(P_i^a - P_i^b\right)$, are introduced, P_i^a and P_i^b are the polarization components of two equivalent sublattices "*a*" and "*b*" [7]. As usual for proper and incipient ferroelectrics, the coefficients a_k are temperature dependent and obeys the Barrett law, $a_k^{(P)}(T) = \alpha_T\left[T_{qP}\coth\left(T_{qP}/T\right) - T_C\right]$, where T_C is the Curie temperature, and T_{qP} is a characteristic temperature [6, 8, 9]. Numerical values of the phenomenological coefficients a_i, a_{ij}, a_{ijk} and gradient coefficients g_{ij} included in Eq.(SI.2b) can be found in Supplementary Table 1.

The compact form of the biquadratic coupling energy between polarization and tilt is

$$\Delta G_{BQC} = \xi_{ijkl}^{aa}\left(P_iP_j + A_iA_j\right)\Phi_k\Phi_l + \xi_{ijkl}^{ab}\left(P_iP_j - A_iA_j\right)\Phi_k\Phi_l, \quad \text{(SI.2c)}$$

where poorly known tensorial AFD-FE biquadratic coupling coefficients, ξ_{ijkl}^{aa} and ξ_{ijkl}^{ab}, are usually treated as fitting parameters to experiment.

Electrostriction and rotostriction contributions are

$$\Delta G_{ST} = -Q_{ijkl}^{aa}\sigma_{ij}(P_kP_l + A_kA_l) - Q_{ijkl}^{ab}\sigma_{ij}(P_kP_l - A_kA_l) - R_{ijkl}\sigma_{ij}\Phi_k\Phi_l, \quad \text{(SI.2d)}$$

where σ_{ij} are elastic stress tensor components, which satisfy the equation of mechanical equilibrium, $\frac{\partial \sigma_{ij}}{\partial x_j} = 0$. Electrostriction and rotostriction coefficients are, Q_{ijkl}^{aa}, Q_{ijkl}^{ab} and R_{ijkl}, respectively. Elastic and flexoelectric contributions are

$$\Delta G_{EL} = -\frac{1}{2} s_{ijkl}\sigma_{ij}\sigma_{kl} - \frac{1}{2} F_{ijkl}\left(\sigma_{ij}\frac{\partial P_k}{\partial x_l} - P_k\frac{\partial \sigma_{ij}}{\partial x_l}\right) + V_{ij}\sigma_{ij}N_d \qquad \text{(SI.2e)}$$

Here s_{ijkl} are the components of elastic compliances tensor (see e.g. Ref.[[10]]); F_{ijkl} are flexoelectric tensor components. The last term in Eq. (SI.2e) is the chemical expansion due to the appearance of elastic defects, i.e. R-impurity with concentration N_d, characterized by the Vegard strain tensor V_{ij} [[11], [12]], which value depends on the impurity and, as a rule, varied in the range (–5 – +5) 10^{-29} m^3. The full form of expressions (SI.2) depends on the concrete form of parent phase symmetry.

For a spatially uniform (i.e. domain-free) system, the equilibrium equations for the long-range order parameters can be found as partial derivatives of Eq.(SI.1), $\frac{\partial G}{\partial \Phi_i} = 0$, $\frac{\partial G}{\partial P_i} = 0$, and $\frac{\partial G}{\partial A_i} = 0$. These equations should be solved along with the equation of state for elastic stress, $\frac{\partial G}{\partial \sigma_{ij}} = -u_{ij}$, where u_{ij} is an elastic strain.

For a general case of inhomogeneous (e.g. domain structured or/and spatially modulated) system, one should solve the coupled Euler-Lagrange equations of states, which are expressed via the variational derivatives of the functional (1):

$$\frac{\delta G}{\delta \Phi_i} = 0, \qquad \frac{\delta G}{\delta P_i} = -E_i, \qquad \frac{\delta G}{\delta A_i} = 0. \qquad \text{(SI.3a)}$$

which solution is equivalent to the minimization of the free energy functional (SI.1), that defines the distribution of the order parameter fields given the boundary conditions on the external surfaces. External and depolarization fields, E_i^{ext} and E_i^d, which contribute to the electric field, $E_i = E_i^{ext} + E_i^d$, can be found from electrostatic equation div**D**=0 with boundary conditions at the surfaces, interfaces and/or electrodes. Elastic fields, which are, in fact, the secondary order parameters, satisfy equation of state and mechanical equilibrium equations:

$$\frac{\partial G}{\partial \sigma_{ij}} = -u_{ij}, \qquad \frac{\partial \sigma_{ij}}{\partial x_j} = 0\,. \qquad \text{(SI.3b)}$$

The strain (or stress) should be defined at the system boundaries.

Minimization of the LGD free energy functional with respect to polarization components P_i leads to three coupled Euler-Lagrange equations for polarization components:

$$2P_1(a_1 - Q_{12}(\sigma_{22} + \sigma_{33}) - Q_{11}\sigma_{11}) - Q_{44}(\sigma_{12}P_2 + \sigma_{13}P_3) + 4a_{11}P_1^3 + 2a_{12}P_1(P_2^2 + P_3^2) + 6a_{111}P_1^5 + 2a_{112}P_1(P_2^4 + 2P_1^2P_2^2 + P_3^4 + 2P_1^2P_3^2) + 2a_{112}P_1P_2^2P_3^2 - g_{11}\frac{\partial^2 P_1}{\partial x_1^2} - g_{44}\left(\frac{\partial^2 P_1}{\partial x_2^2} + \frac{\partial^2 P_1}{\partial x_3^2}\right) + F_{11}\frac{\partial \sigma_{11}}{\partial x_1} + F_{12}\left(\frac{\partial \sigma_{22}}{\partial x_1} + \frac{\partial \sigma_{33}}{\partial x_1}\right) + F_{44}\left(\frac{\partial \sigma_{12}}{\partial x_2} + \frac{\partial \sigma_{13}}{\partial x_3}\right) = E_1 \quad \text{(SI.4a)}$$

$$2P_2(a_1 - Q_{12}(\sigma_{11} + \sigma_{33}) - Q_{11}\sigma_{22}) - Q_{44}(\sigma_{12}P_1 + \sigma_{23}P_3) + 4a_{11}P_2^3 + 2a_{12}P_2(P_1^2 + P_3^2) + 6a_{111}P_2^5 + 2a_{112}P_2(P_1^4 + 2P_2^2P_1^2 + P_3^4 + 2P_2^2P_3^2) + 2a_{112}P_2P_1^2P_3^2 - g_{11}\frac{\partial^2 P_2}{\partial x_2^2} - g_{44}\left(\frac{\partial^2 P_2}{\partial x_1^2} + \frac{\partial^2 P_2}{\partial x_3^2}\right) + F_{11}\frac{\partial \sigma_{22}}{\partial x_2} + F_{12}\left(\frac{\partial \sigma_{11}}{\partial x_2} + \frac{\partial \sigma_{33}}{\partial x_2}\right) + F_{44}\left(\frac{\partial \sigma_{12}}{\partial x_1} + \frac{\partial \sigma_{23}}{\partial x_3}\right) = E_2 \quad \text{(SI.4b)}$$

$$2P_3(a_1 - Q_{12}(\sigma_{11} + \sigma_{22}) - Q_{11}\sigma_{33}) - Q_{44}(\sigma_{13}P_1 + \sigma_{23}P_2) + 4a_{11}P_3^3 + 2a_{12}P_3(P_1^2 + P_2^2) + 6a_{111}P_3^5 + 2a_{112}P_3(P_1^4 + 2P_3^2P_1^2 + P_2^4 + 2P_2^2P_3^2) + 2a_{112}P_3P_1^2P_2^2 - g_{11}\frac{\partial^2 P_3}{\partial x_3^2} - g_{44}\left(\frac{\partial^2 P_3}{\partial x_1^2} + \frac{\partial^2 P_3}{\partial x_2^2}\right) + F_{11}\frac{\partial \sigma_{33}}{\partial x_3} + F_{12}\left(\frac{\partial \sigma_{11}}{\partial x_3} + \frac{\partial \sigma_{33}}{\partial x_3}\right) + F_{44}\left(\frac{\partial \sigma_{13}}{\partial x_1} + \frac{\partial \sigma_{23}}{\partial x_2}\right) = E_3 \quad \text{(SI.4c)}$$

As usual, the coefficient a_i linearly depends on temperature T, $a_i(T) = \alpha_T[T - T_C\ ]$. Tensor components a_{ij} and a_{ijk} are regarded temperature-independent. Tensor a_{ij} is positively defined if the ferroelectric material undergoes a second order transition to the paraelectric phase and negative otherwise. Higher nonlinear tensor a_{ijk} and gradient coefficients tensor g_{ijkl} are positively defined and regarded as temperature independent. The value σ_{ij} is the elastic stress tensor and s_{ijkl} is the compliances tensor, Q_{ijkl} is electrostriction tensor, and F_{ijkl} is the flexoelectric tensor. As argued by Hlinka et al. [13], it is reasonable to assume that $g'_{44} = -g_{12}$, and corresponding terms are omitted in Eqs.(SI.4).

The boundary condition for polarization at the ferroelectric surface S accounts for the flexoelectric effect:

$$\left(g_{ijkl}\frac{\partial P_k}{\partial x_l} - F_{klij}\sigma_{kl}\right)n_j\Big|_S = 0 \quad \text{(SI.5a)}$$

where **n** is the outer normal to the surface S, i=1, 2, 3.

Electric field components E_i are related to the electric potential φ in a conventional way, $E_i = -\partial\varphi/\partial x_i$. The potential φ satisfies the Poisson equation in the ferroelectric:

$$\varepsilon_0\varepsilon_b\left(\frac{\partial^2}{\partial x_1^2} + \frac{\partial^2}{\partial x_2^2} + \frac{\partial^2}{\partial x_3^2}\right)\varphi = \frac{\partial P_i}{\partial x_i}, \quad \text{(SI.5b)}$$

where ε_b is a relative permittivity of the background [14], and ε_0 is a universal dielectric constant. The boundary conditions to Eq.(SI.5) depend on the ferroelectric geometry and electrodes configuration. For many cases the condition $div\mathbf{E} = 0$ is consistent with $\mathbf{E} = 0$, indicating the formation of the nominally uncharged domain structure.

At first, we consider a hypothetic situation of the stress-free system with one-, two- or three-component polarization, $P_1(x'_3)$, $P_2(x'_3)$ and $P_3(x'_3)$, when the coupled time-dependent Euler-Lagrange equations acquire much simpler form:

$$2a_1P_1 + 4a_{11}P_1^3 + 2a_{12}P_1(P_2^2 + P_3^2) + 6a_{111}P_1^5 + 2a_{112}P_1(P_2^4 + 2P_1^2P_2^2 + P_3^4 + 2P_1^2P_3^2) + 2a_{112}P_1P_2^2P_3^2 - g_{44}\frac{\partial^2 P_1}{\partial x_3'^2} = 0 \quad \text{(SI.6a)}$$

$$2a_1P_2 + 4a_{11}P_2^3 + 2a_{12}P_2(P_1^2 + P_3^2) + 6a_{111}P_2^5 + 2a_{112}P_2(P_1^4 + 2P_2^2P_1^2 + P_3^4 + 2P_2^2P_3^2) + 2a_{112}P_2P_1^2P_3^2 - g_{44}\frac{\partial^2 P_2}{\partial x_3'^2} = 0 \quad \text{(SI.6b)}$$

$$2a_1P_3 + 4a_{11}P_3^3 + 2a_{12}P_3(P_1^2 + P_2^2) + 6a_{111}P_3^5 + 2a_{112}P_3(P_1^4 + 2P_3^2P_1^2 + P_2^4 + 2P_2^2P_3^2) + 2a_{112}P_3P_1^2P_2^2 - g'_{11}\frac{\partial^2 P_3}{\partial x_3'^2} = 0 \quad \text{(SI.6c)}$$

The coordinate x'_3 coincides with x_3 for the one- polarization components, $P_1(x_3)$, or two-, $P_2(x_3)$ and $P_3(x_3)$, respectively. For a three-component case, the coordinate frame should be rotated, and after this x'_3 becomes perpendicular to **P** vector, in order to remain the domain walls uncharged. All other tensors should be rotated also.

Nonlinear coupled equations (SI.6) can be solved numerically for concrete boundary conditions (SI.4), which explicit form is:

$$\left.\left(a_1^S P_1 + g_{44}\frac{\partial P_1}{\partial x'_3}n'_3\right)\right|_S = 0, \quad \left.\left(a_2^S P_2 + g_{44}\frac{\partial P_2}{\partial x'_3}n'_3\right)\right|_S = 0, \quad \left.\left(a_3^S P_3 + g'_{11}\frac{\partial P_3}{\partial x'_3}n'_3\right)\right|_S = 0 \quad \text{(SI.7)}$$

Analytical solution of nonlinear the system (SI.6) is possible in several cases for very specific boundary conditions, but not for more general conditions (SI.4).

Analytical solutions:

One exclusion is the so-called periodic *a*-domain structure imposed to the natural boundary conditions, $\left.\frac{\partial P_i}{\partial x'_3}n'_3\right|_S = 0$, where *i*=1, 2, 3. Using the following re-designation $P_1 = \eta$ and $P_{2,3} = 0$ (e.g. in tetragonal case); or $P_1 = P_2 = P$ and $P_3 = 0$ (e.g. in orthorhombic phase); or $P_1 = P_2 = P_3 = P$ (e.g. in rhombohedral phase), formally Eqs.(SI.6) reduces to one equation:

$$2\alpha P + 4\beta P^3 + 6\gamma P^5 - g\frac{\partial^2 P}{\partial x_3'^2} = 0 \quad \text{(SI.8a)}$$

With a boundary condition $\left.\frac{\partial \eta}{\partial x'_3}n'_3\right|_{\pm\frac{h}{2}} = 0$. Parameters $\alpha\sim a_1$, $\beta\sim a_{11}$, and $\gamma\sim a_{111}$. The analytical solution is:

$$P(x) = P_b\sqrt{\frac{2m}{1+m}}\,sn\left(\frac{x-x_0}{L_c\sqrt{1+m}}\middle|\, m\right) \quad \text{(SI.8b)}$$

Here $P_b = \sqrt{-\alpha/\beta}$ and $L_C = \sqrt{g/(-\alpha)}$. Also we put $\gamma = 0$.

However, the *a*-solution cannot describe mixed (e.g. Bloch-Ising) domain walls.

Next let us consider the case of a *single domain wall*.

Case I. Static one-component and one-dimensional partial solution. If only the one component of polarization is coordinate dependent, and another component is zero, the one-dimensional profile of uncharged domain wall is given by expression

$$P_2(x_3) = \frac{P_S \cdot \tanh[(x_3 - x_0)/L_c]}{\sqrt{\eta \cdot \mathrm{sech}^2[(x_3 - x_0)/L_c] + 1}}, \qquad P_1(x_3) = 0, \qquad \text{(SI.8c)}$$

where P_S is the spontaneous polarization, $x_2 - x_0$ is the distance from center of the domain wall plane, and 2 R_c is the domain wall width [15]. For the second-order ferroelectrics $P_S^2 = -a_1/(2a_{11})$ and $\eta = 0$, while for the fist-order ferroelectrics $P_S^2 = \left(\sqrt{a_{11}{}^2 - 4a_1a_{111}} - a_{11}\right)/2a_{111}$ and dimensionless parameter $\eta = a_{111}P_S^2/(a_{11} + 2a_{111}P_S^2)$ is positive. The correlation length $L_c = \sqrt{g_{44}/(a_1 + 3a_{11}P_S^2 + 5a_{111}P_S^4)}$. The expression (SI.8) describes 180-degree Ising-type uncharged domain wall (see Supplementary Figure 2).

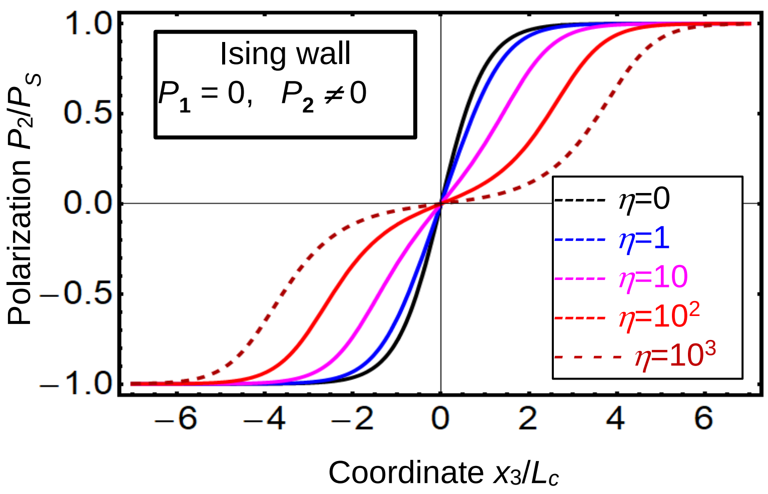

Supplementary Figure 2. Distribution of polarization component P_2/P_S across the domain wall. Polarization profile given by Eq.(SI.5) for different values of η=0 (black curve), 1 (blue curve), 10 (magenta curve), 10^2 (red curve) and 10^4 (dashed curve).

As one can see the boundary condition (SI.7) is incompatible with the solution (SI.8) in a general case. However, a rather small incompatibility corresponds to the specific case $a^{(S)} = 0$, $|x_0| \ll L$ and $L \gg R_c$, where L in the characteristic size in x_3 direction, since $\mathrm{sech}^2[(L - x_0)/L_c] \ll 1$ for the case. Allowing for R_c has an order of lattice constant, and the core radius should be not less

than (10 – 20) lattice constants for the applicability of continuous LGD approach, the solution (SI.8) can be considered as a relevant trial function for the case $a^{(S)} = 0$.

Case II. Static two-component and one-dimensional partial solution. For the ferroelectrics with the second order phase transition ($a_{11} > 0$ and $a_{111} = a_{112} = 0$) the one-dimensional profile of uncharged domain walls satisfies the simplified Eqs.(SI.6), which can be as following:

$$2a_1 P_1 + 4a_{11} P_1^3 + 2a_{12} P_1 P_2^2 - g_{44} \frac{\partial^2 P_1}{\partial x_3^2} = 0\,, \tag{SI.9a}$$

$$2a_1 P_2 + 4a_{11} P_2^3 + 2a_{12} P_2 P_1^2 - g_{44} \frac{\partial^2 P_2}{\partial x_3^2} = 0\,. \tag{SI.9b}$$

For the specific case $a_{12} = 6a_{11}$, the partial solution of Eqs.(SI.9) is [16]:

$$P_1(x_3) = \frac{P_S \cdot \sinh[R_0/L_c]}{\cosh[R_0/L_c] + \cosh[(x_3 - x_0)/L_c]}, \quad P_2(x_3) = \frac{P_S \cdot \sinh[(x_3 - x_0)/L_c]}{\cosh[R_0/L_c] + \cosh[(x_3 - x_0)/L_c]}. \tag{SI.10}$$

where $P_S = \sqrt{-a_1/(2a_{11})}$ is the spontaneous polarization ($a_1 < 0$), $x_3 - x_0$ is the distance from center of the domain wall plane, the correlation length is $L_c = \sqrt{-g_{44}/(2a_1)}$, and R_0 is an arbitrary constant. For the particular case $R_0 = 0$ we obtain $P_1(x_3) = 0$ and $P_2(x_3) = P_S \tanh[(x_3 - x_0)/L_c]$, i.e. the solution (SI.8) for the second order phase transitions. At nonzero R_0 the profile (SI.10) is an uncharged Ising-Bloch type domain wall (two "rotational" 180-degree c-domains separated by an a-domain, see Supplementary Figure 3). Note that the wall energy is R_0-independent [16].

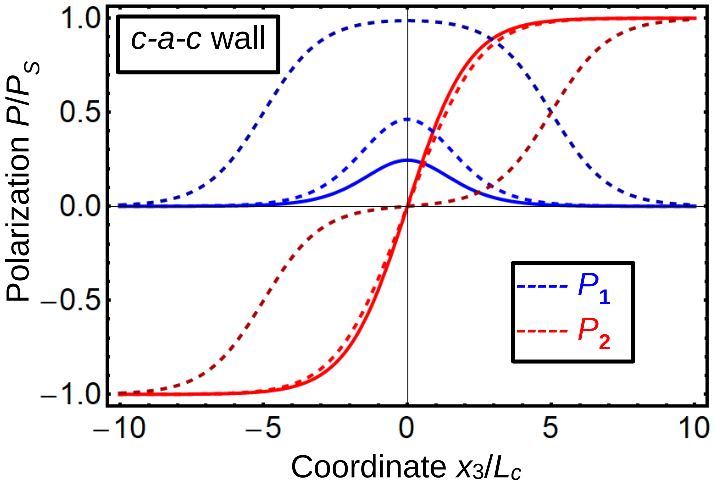

Supplementary Figure 3. Distribution of polarization components. P_1/P_S (blue curves) and P_2/P_S (red curves), across the domain wall, given by Eq.(SI.10) for different values of R_0/L_c=0.5 (solid curves), 1 (dashed curves) and 5 (dashed darker curves).

Note that the boundary condition (SI.5a) imposed the ferroelectric surface affects weakly on the solution (SI.10) for the specific case $a^{(S)} = 0$, $|x_0| \ll L$ and $L \gg L_c$. Assuming that these inequalities are valid, the solution (SI.10) can be considered as a relevant trial function.

Since both partial solutions (SI.8) and (SI.10) are expressed via hyperbolic functions, the static solution of nonlinear coupled Eqs.(SI.6) can be found by a variational principle using principle using hyperbolic functions as a basis for serial expansion for trial functions:

$$P_2(x_3) = P_a \tanh\left(\frac{x_3 - x_a}{a}\right) + P_b \tanh\left(\frac{x_3 - x_b}{b}\right), \qquad \text{(SI.11a)}$$

$$P_2(x_3) = P_a \tanh\left(\frac{x_3 - x_a}{a}\right) - P_b \tanh\left(\frac{x_3 - x_b}{b}\right) + P_c\left[1 - \mu\, cosh^{-2}\left(\frac{x_3 - x_c}{c}\right)\right], \qquad \text{(SI.11b)}$$

where $P_{a,b,c}$, $x_{a,b,c}$, a, b, c and μ are variational parameters.

Analysis of the phase portrait and other solutions for arbitrary $-2a_{11} < a_{12}$:

Introducing the dimensionless variables and order parameters,

$$x = \frac{x_3}{R_c}, \qquad p_1 = \frac{P_1}{P_S}, \qquad p_2 = \frac{P_2}{P_S}, \qquad \mu = \frac{a_{12}}{2a_{11}} \qquad \text{(SI.16)}$$

where $P_S = \sqrt{-a_1/(2a_{11})}$ and $R_c = \sqrt{-g_{44}/(2a_1)}$, one could get the coupled Euler-Lagrange equations:

$$\frac{\partial^2}{\partial x^2} p_1 = -p_1 + p_1^3 + \mu p_1 p_2^2, \qquad \text{(SI.17a)}$$

$$\frac{\partial^2}{\partial x^2} p_2 = -p_2 + p_2^3 + \mu p_2 p_1^2. \qquad \text{(SI.17b)}$$

Equations (SI.17) depends on the only parameter $-1 < \mu$. The free energy density and the first integral are

$$g_{LGD} = -\frac{1}{2}(p_1^2 + p_2^2) + \frac{1}{4}(p_1^4 + p_2^4) + \frac{\mu}{2} p_1^2 p_2^2 + \frac{1}{2}\left[\left(\frac{dp_1}{dx}\right)^2 + \left(\frac{dp_2}{dx}\right)^2\right], \qquad \text{(SI.18a)}$$

$$I_1 = -\frac{1}{2}(p_1^2 + p_2^2) + \frac{1}{4}(p_1^4 + p_2^4) + \frac{\mu}{2} p_1^2 p_2^2 - \frac{1}{2}\left[\left(\frac{dp_1}{dx}\right)^2 + \left(\frac{dp_2}{dx}\right)^2\right]. \qquad \text{(SI.18b)}$$

Several numerical solutions of Eqs.(SI. 17) are shown in Supplementary Figure 4 for several values of the parameter μ.

After elementary transformations, the first integral and free energy density become:

$$I_1[\mu] = -\frac{1}{4}\frac{\partial^2}{\partial x^2}(p_1^2 + p_2^2) - (p_1^2 + p_2^2) + \frac{3}{4}(p_1^2 + p_2^2)^2 + \frac{3}{2}(\mu - 1) p_1^2 p_2^2. \qquad \text{(SI.18c)}$$

Equation (18c) account for the identities, $\frac{\partial^2}{\partial x^2} p_1^2 = 2p_1 \frac{\partial^2 p_1}{\partial x^2} + 2\left(\frac{dp_1}{dx}\right)^2$ and $p_1 \frac{\partial^2 p_1}{\partial x^2} + p_2 \frac{\partial^2 p_2}{\partial x^2} = -(p_1^2 + p_2^2) + (p_1^4 + p_2^4) + 2\mu p_1^2 p_2^2$, obtained from Eqs.(SI.17). Two homogeneous phases are consistent with Eq.(SI.18a):

$$p_1 = p_2 = \pm\frac{1}{\sqrt{1+\mu}}, \; g_{LGD} = -\frac{1}{2(1+\mu)}, \; I_1[\mu] = -\frac{1}{2(1+\mu)} \quad \text{stable at } -1 < \mu < 1, \qquad \text{(SI.19a)}$$

$$p_1^2 = 0, \; p_2^2 = 1, \text{ or } p_1^2 = 1, \; p_2^2 = 0, \; g_{LGD} = -\frac{1}{4}, \; I_1[\mu] = -\frac{1}{4} \text{ stable at } \mu > 1, \qquad \text{(SI.19b)}$$

Results of FEM simulations are shown in Supplementary Figure 4, their form is very close to the one given by Eqs.(SI.20).

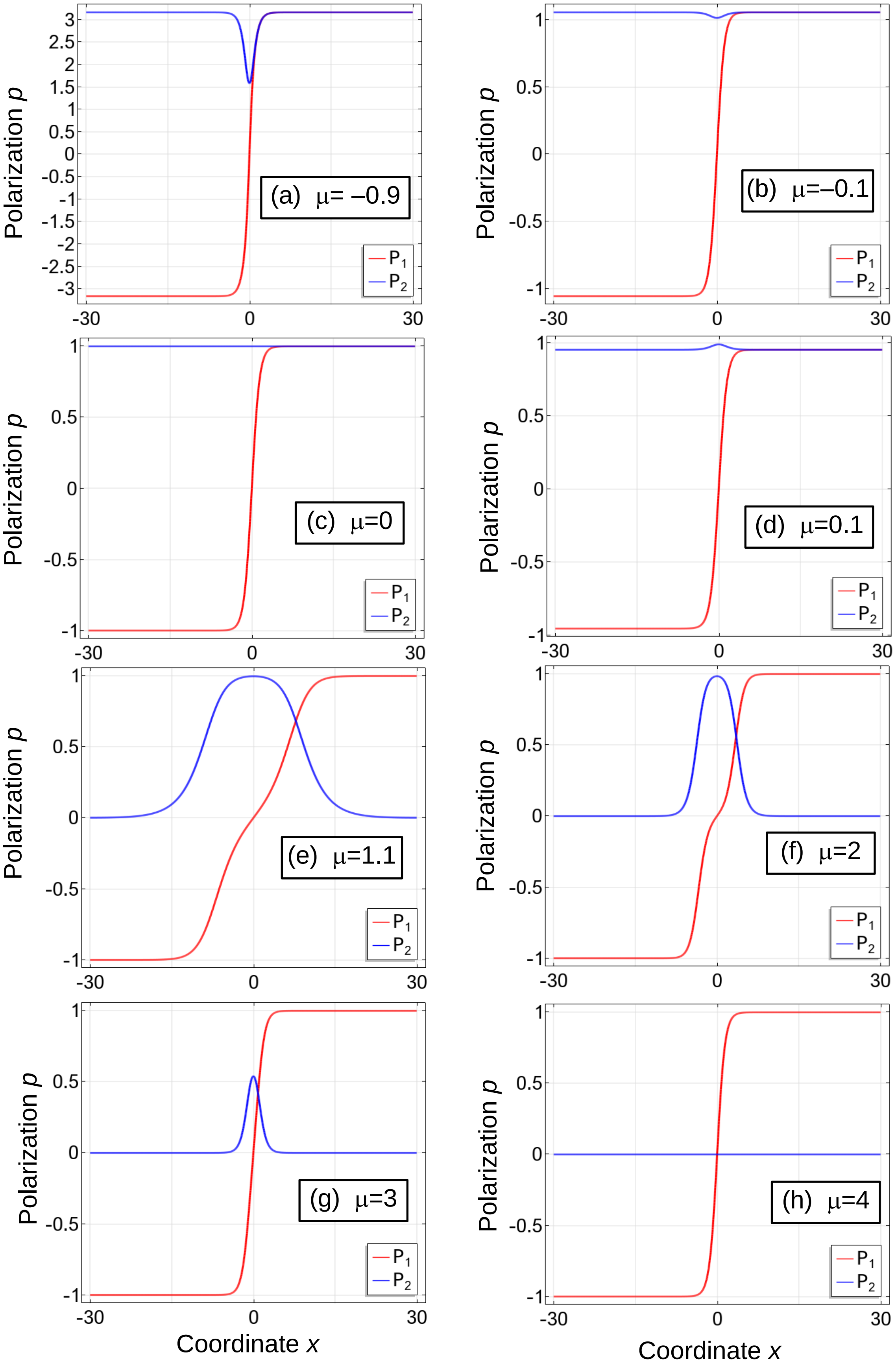

Supplementary Figure 4. Distributions of polarization components vs. μ. P_1/P_S (red curves) and P_2/P_S (blue curves) calculated by FEM for different values of μ =-0.9 (a), -0.1 (b), 0 (c), 0.1 (d), 1.1 (e), 2 (f), 3(g) and 4 (h).

Direct variational method can be applied for the trial functions (SI.11) written in dimensionless form:

$$p_1(x) = p_a \tanh\left(\frac{x+x_b}{b}\right) + p_b \tanh\left(\frac{x-x_b}{b}\right), \qquad \text{(SI.20a)}$$

$$p_2(x) = p_a \tanh\left(\frac{x+x_b}{b}\right) - p_b \tanh\left(\frac{x-x_b}{b}\right) + c\left[1 - p_c\, cosh^{-2}\left(\frac{x-x_b}{b}\right)\right], \qquad \text{(SI.20b)}$$

where p_a, p_b, x_b and b are variational parameters, which can be determined after substitution of Eqs.(SI.20) in the free energy (SI.18a), integration $\frac{1}{L}\int_{-L}^{L} g_{LGD}(x)dx = min$ and minimization over them.

Case III. Role of the flexoelectric coupling. Approximate analytical expressions for polarization components $\tilde{P}_i(\tilde{x}_1)$ in the vicinity of uncharged 180, 109 and 71 degree ferroelectric domain walls in the rhombohedral phase of multiaxial ferroelectric allowing for the electrostriction and flexoelectric coupling have been derived in Ref.[17].

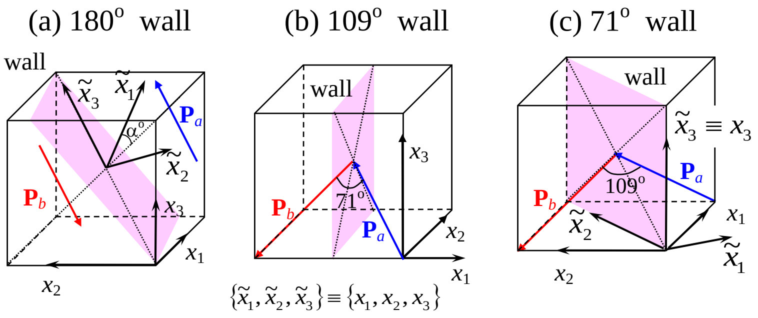

Supplementary Figure 5. Rotated coordinate frame $\{\tilde{x}_1, \tilde{x}_2, \tilde{x}_3\}$ choice for (a) 180°, (b) 109° and (c) 71° uncharged domain walls in a rhombohedral ferroelectric. Pseudo-cubic crystallographic axes are $\{x_1, x_2, x_3\}$. Domain wall rotation angle α is counted from $\tilde{x}_3$ axes.

Supplementary Table 1 summarizes these results and suggests more general trial functions. Fitting constants f_1^Q and f_2^Q are proportional to the flexocoupling tensor components, the constants q_1 and q_2 are proportional to the electrostriction tensor components. Note that the Neel-type component $\tilde{P}_1$ is typically rather small as being affected by depolarization field. However, the appearance of the component is a distinct feature of flexoelectric coupling

Supplementary Table 1. Approximate analytical expressions for polarization components $\tilde{P}_i(\tilde{x}_1)$ in the vicinity of uncharged domain walls in the rhombohedral phase of $BiFeO_3$.

Domain wall (DW)	109 degree DW	71 degree DW	180 degree DW

**Rotation angle α**	α = 0, π in the **rhombohedral** phase DW is absent in the **tetragonal** phase	α = −π/4, 3π/4 in the **rhombohedral** phase DW is absent in the **tetragonal** phase	α is arbitrary in both **rhombohedral** and **tetragonal** phases
**Compo-nent** $\tilde{P}_3$	$\tilde{P}_3^S \tanh\left(\frac{\tilde{x}_1}{L_c}\right)$	$\tilde{P}_3^S \tanh\left(\frac{\tilde{x}_1}{L_c}\right)$	$\tilde{P}_3^S \tanh\left(\frac{\tilde{x}_1}{L_c}\right)$
**Compo-nent** $\tilde{P}_2$ **	$\tilde{P}_2^S \tanh\left(\frac{\tilde{x}_1}{L_c}\right)$	0	$P_B \operatorname{sech}^2\left(\frac{\tilde{x}_1}{L_c}\right) + f_2^Q \frac{\partial \tilde{P}_3^2}{\partial \tilde{x}_1} + q_2 \tilde{P}_3\left(\left(\tilde{P}_3^S\right)^2 - \tilde{P}_3^2\right)$
**Compo-nent** $\tilde{P}_1$	$\tilde{P}_1^S + f_1^Q\left(\frac{\partial \tilde{P}_3^2}{\partial \tilde{x}_1} + \frac{\partial \tilde{P}_2^2}{\partial \tilde{x}_1}\right)$	$\tilde{P}_1^S + f_1^Q \frac{\partial \tilde{P}_3^2}{\partial \tilde{x}_1}$	$f_1^Q \frac{\partial \tilde{P}_3^2}{\partial \tilde{x}_1} + q_1 \tilde{P}_3\left(\left(\tilde{P}_3^S\right)^2 - \tilde{P}_3^2\right)$

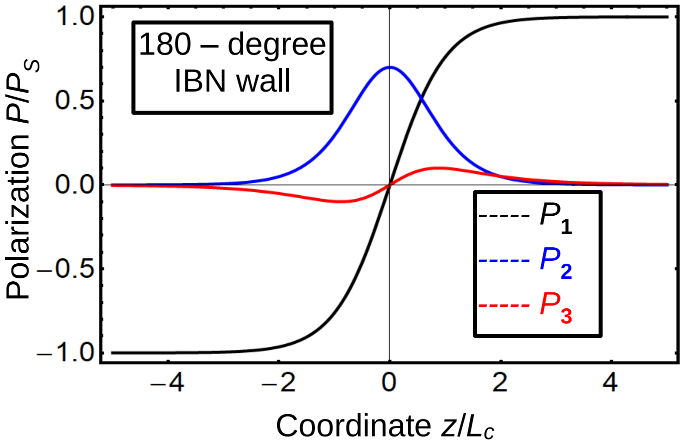

Supplementary Figure 6. Distribution of polarization components across the mixed type Ising-Bloch-Neel (IBN) domain wall. P_i/P_S (black, red and blue curves), given by Eq.(SI.12) for different amplitudes P_B/P_S=0.7 for Bloch component P_2, and P_N/P_S=0.2 for Neel component P_3.

The generalization of results listed in Supplementary Table 1 has the form:

$$P_i(\mathrm{z}) = \sum_{j=1}^{m}\left(\tanh\left(\frac{z-z_{ij}}{L_{ij}}\right)\left[a_{ij} + f_{ij}\operatorname{sech}^2\left(\frac{z-z_{ij}}{L_{ij}}\right)\right] + q_{ij}\operatorname{sech}^2\left(\frac{z-z_{ij}}{L_{ij}}\right)\right). \qquad \text{(SI.12)}$$

Here $i = 1,2,3$. The fitting parameters a_{ij}, f_{ij} and q_{ij} are amplitudes related with the spontaneous value (ferroelectric nonlinearity), flexoelectric and electrostriction couplings, respectively, and L_{ij} are correlation lengths.

Supplementary Table 2. Parameters used in FEM for BFO collected from Refs. [18, 19, 20]

**Parameter**	**Designation**	**Numerical values for BFO (BFO:R)**	**Ref**
Effective permittivity	$\varepsilon_{eff} = \Sigma_i \varepsilon_{bi} + \varepsilon_{el}$	7	f.p.
dielectric stiffness	α_T ($\times 10^5 C^{-2}\cdot$Jm/K)	9	[18]
Curie temperature for P	T_C (K)	1300	[18]
Barret temperature for P	T_{qP} (K)	800	[18]

polar expansion 4th order	a_{ij} (×$10^8$$C^{-4}$·$m^5$J)	a_{11}= −13.5, a_{12}= 5	[18]
LGD expansion 6th order	a_{ijk} (×$10^9$$C^{-6}$·$m^9$J)	a_{111}= 11.2, a_{112}= −3, a_{123}= −6	[19]
electrostriction	Q_{ij} (C^{-2}·m^4)	Q_{11}=0.054, Q_{12}= −0.015, Q_{44}=0.02	[20]
Stiffness components	c_{ij} (×10^{11} Pa)	c_{11}=3.02, c_{12}= 1.62, c_{44}=0.68	f.p.
polarization gradient coefficients	g_{ij} (×$10^{-10}$$C^{-2}$$m^3$J)	BFO – g_{11}=8, g_{12}= −0.5, g_{44}=5	[18]
AFD-FE coupling	ξ_{ij} (×10^{29} C^{-2}·m^{-2} J/K)	ξ_{11} = −0.5, ξ_{12} =0.5, ξ_{44} = −2.6	[18]
tilt expansion 2nd order	b_T (×10^{26}·J/(m^5K))	4	[18]
Curie temperature for Φ	T_Φ (K)	1440	[18]
Barret temperature for Φ	$T_{q\Phi}$ (K)	400	[18]
tilt expansion 4nd order	b_{ij} (×10^{48}J/m^7)	b_{11}= −24+4.5 $(\coth(300/T)-\coth(3/14))$<br>$b_{12}$= 45−4.5 $(\coth(300/T)-\coth(1/4))$	[18]
tilt expansion 6nd order	b_{ijk} (×10^{70} J/m^9)	b_{111}= 4.5−3.4 $(\coth(400/T)-\coth(2/7))$<br>$b_{112}$= 3.6−0.04 $(\coth(10/T)-\coth(1/130))$<br>$b_{123}$= 41−43.2 $(\coth(1200/T)-\coth(12/11))$	f.p.
tilt gradient coefficients	v_{ij} (×10^{11} J/ m^3)	v_{11}=2, v_{12}= −1, v_{44}=1	[19]
rotostriction	R_{ij} (×10^{18} m^{-2})	R_{11}= −1.32, R_{12}= −0.43, R_{44}=8.45	f.p.
Flexoelectric coefficients	F_{ij} (×10^{-11} m^3/C)	F_{11}= 2, F_{12}= 1, F_{44}= 0.5	f.p.

Supplementary References:

1 I. O. Troyanchuk, D. V. Karpinsky, M. V. Bushinsky, O. S. Mantytskaya, N. V. Tereshko, and V. N. Shut. "Phase Transitions, Magnetic and Piezoelectric Properties of Rare-Earth-Substituted BiFeO 3 Ceramics." *Journal of the American Ceramic Society* **94**, no. 12 (2011): 4502-4506.

2 A.Y. Borisevich, E.A. Eliseev, A.N. Morozovska, C.-J. Cheng, J.-Y. Lin, Y.H. Chu, D. Kan, I. Takeuchi, V. Nagarajan, S.V. Kalinin. Atomic-scale evolution of modulated phases at the ferroelectric–antiferroelectric morphotropic phase boundary controlled by flexoelectric interaction. Nature Communications. **3**, 775 (2012).)

3 R. Maran, S. Yasui, E.A. Eliseev, M.D. Glinchuk, A.N. Morozovska, H. Funakubo, I. Takeuchi, N. Valanoor. Interface control of a morphotropic phase boundary in epitaxial samarium-modified bismuth ferrite superlattices. Phys. Rev. B **90**, 245131 (2014)

4 R. Maran, S. Yasui, E. Eliseev, A. Morozovska, F. Hiroshi, T. Ichiro, V. Nagarajan. "Enhancement of dielectric properties in epitaxial bismuth ferrite – bismuth samarium ferrite superlattices" Adv. Electron. Mater., **2**, 1600170 (2016). DOI: 10.1002/aelm.201600170

5 E. A. Eliseev, A. N. Morozovska, C. T. Nelson, and S. V. Kalinin, Intrinsic structural instabilities of domain walls driven by gradient coupling: Meandering antiferrodistortive-ferroelectric domain walls in $BiFeO_3$. Phys. Rev. B **99**, 014112 (2019).

6 J.H. Barrett, Dielectric Constant in Perovskite Type Crystals, Phys. Rev. **86**, 118-120 (1952).

7 C. Kittel, Theory of antiferroelectric crystals, Phys. Rev. **82**, 729 (1951).

8 D.V. Karpinsky, E.A. Eliseev, F. Xue, M.V. Silibin, A. Franz, M.D. Glinchuk, I.O. Troyanchuk, S.A. Gavrilov, V. Gopalan, L.-Q. Chen, and A.N. Morozovska, Thermodynamic potential and phase diagram for multiferroic bismuth ferrite ($BiFeO_3$), npj Computational Materials **3**, 20 (2017).

9 A.N. Morozovska, E.A. Eliseev, M.D. Glinchuk, O.M. Fesenko, V.V. Shvartsman, V. Gopalan, M.V. Silibin, and D.V. Karpinsky, Rotomagnetic coupling in fine-grained multiferroic $BiFeO_3$: Theory and experiment, Phys. Rev. B **97**, 134115 (2018).

10 J.X. Zhang, Y.L. Li, Y. Wang, Z.K. Liu, L.Q. Chen, Y.H. Chu, F. Zavaliche, and R. Ramesh, Effect of substrate-induced strains on the spontaneous polarization of epitaxial BiFeO3 thin films, J. Appl. Phys. **101**, 114105 (2007).

11 D. A. Freedman, D. Roundy, and T. A. Arias, Elastic effects of vacancies in strontium titanate: Short-and long-range strain fields, elastic dipole tensors, and chemical strain, Phys. Rev. B 80, 064108 (2009).

12 A. N. Morozovska, E. A. Eliseev, M. D. Glinchuk, R. Vasudevan, M. V. Silibin, Y. A. Genenko and S. V. Kalinin. Mesoscopic theory of defect ordering-disordering transitions in thin oxide films (http://arxiv.org/abs/1911.00258)

13 J. Hlinka and P. Márton, Phenomenological model of 90-degree domain wall in $BaTiO_3$ type ferroelectrics. Phys. Rev. B **74**, 104104 (2006).

14 A. K. Tagantsev and G. Gerra, Interface-induced phenomena in polarization response of ferroelectric thin films, J. Appl. Phys. **100**, (2006) 051607

15 R. K Behera, C.-W. Lee, D. Lee, A. N Morozovska, S. B Sinnott, A. Asthagiri, V. Gopalan, and S. R Phillpot. Structure and energetics of 180° domain walls in $PbTiO_3$ by density functional theory. *Phys.: Condens. Matter*, **23** 175902 (2011)

16 I.I. Ivanchyk. To macroscopic theory of ferroelectrics. Solid State Physics, **3**, 3731 (1961) (in Russian)

17 A. N. Morozovska, R. K. Vasudevan, P. Maksymovych, S. V. Kalinin and E. A. Eliseev. Anisotropic conductivity of uncharged domain walls in $BiFeO_3$. Phys. Rev. B. **86**, 085315 (2012)

18 D. V. Karpinsky, E. A. Eliseev, F. Xue, M. V. Silibin, A. Franz, M. D. Glinchuk, I. O. Troyanchuk, S. A. Gavrilov, V. Gopalan, L.-Q. Chen, and A. N. Morozovska. "Thermodynamic potential and phase diagram for multiferroic bismuth ferrite ($BiFeO_3$)". npj Computational Materials **3**, 20 (2017)

19 A. N. Morozovska, E. A. Eliseev, M. D. Glinchuk, O. M. Fesenko, V. V. Shvartsman, V. Gopalan, M. V. Silibin, and D. V. Karpinsky. Rotomagnetic coupling in fine-grained multiferroic BiFeO3: Theory and experiment. Phys. Rev. B **97**, 134115 (2018)

20 J. X. Zhang, Y. L. Li, Y. Wang, Z. K. Liu, L. Q. Chen, Y. H. Chu, F. Zavaliche, and R. Ramesh. Effect of substrate-induced strains on the spontaneous polarization of epitaxial $BiFeO_3$ thin films. J. Appl. Phys. **101**, 114105 (2007).
