# Supplementary material for: Exploring physics of ferroelectric domain walls via Bayesian analysis of atomically resolved STEM data
Source: Nat Commun. 2020 Dec 11;11:6361. doi: 10.1038/s41467-020-19907-2 (PMC7733522; doi:10.1038/s41467-020-19907-2)
Supplement: Supplementary file 3 — Description of Additional Supplementary Files [file 41467_2020_19907_MOESM3_ESM.pdf]

Description of Additional Supplementary Files

File Name:Supplementary Data 1
Description: Python notebook of STEM data analysis
